# Survey of ‘*Candidatus* Liberibacter solanacearum’ and Its Potential Psyllid Vectors in Northwestern Italy

**DOI:** 10.3390/insects16050499

**Published:** 2025-05-07

**Authors:** Ahmed Y. S. Oraby, Valentina Candian, Rosemarie Tedeschi

**Affiliations:** Department of Agricultural, Forestry and Food Sciences (DISAFA), University of Turin, Largo P. Braccini 2, Grugliasco, 10095 Turin, Italy; ahmed.oraby@unito.it (A.Y.S.O.); valentina.candian@unito.it (V.C.)

**Keywords:** Psylloidea, *Bactericera trigonica*, *Heterotrioza chenopodii*, *Bactericera nigricornis*, *Trioza urticae*, Apiaceae, Solanaceae, carrot, potato

## Abstract

Psyllids (Hemiptera: Psylloidea) are the main vectors of various phloem-limited plant pathogens, including ‘*Candidatus* Liberibacter’ species. ‘*Candidatus* Liberibacter solanacearum’ (CLso) has been associated with various plant disorders and economic losses in plants from the Solanaceae and Apiaceae families. Recently, it has been reported in Europe, primarily linked to carrots and celery. This situation presents a significant threat, prompting the need for a survey to assess the presence of the bacterium and its potential vectors. Plant and psyllid samples were collected from potato (*Solanum tuberosum*), carrot (*Daucus carota*) and other wild weed species in commercial fields and urban areas over two consecutive years (2022 and 2023). DNA was extracted from the samples, followed by conventional PCR and the sequencing of positive samples. The psyllid species *Bactericera nigricornis* was the dominant species in potato fields, while *Bactericera trigonica* was the most abundant in carrot fields, followed by *Heterotrioza chenopodii*, ranking as the second most abundant species in both cases. CLso-positive samples were found in *D. carota*, *B. trigonica*, *H. chenopodii*, *B. nigricornis*, and *Trioza urticae*. The sequencing results suggest the detected haplotypes are D and U. These findings raise concerns about the potential spread of CLso and the associated risk of significant economic losses.

## Simple Summary

Psyllids are a group of insects responsible for causing economic damage to crops both directly through their feeding behavior and indirectly, transmitting bacterial pathogens that circulate within plant sap from one plant to another. Among these pathogens, ‘*Candidatus* Liberibacter solanacearum’ (CLso) is associated with symptoms on plants of the Apiaceae and Solanaceae families. CLso was first reported in Europe in Finland followed by Norway and several other countries, with cases where it was linked to economic losses in carrot and celery crops. The presence of this bacterium in the region under study was unknown, prompting a survey to assess its presence and investigate potential psyllid vectors. The survey was conducted in Northwestern Italy, specifically in the Piedmont region. The bacterium was detected in carrot plant samples as well as in four psyllid species: *Bactericera trigonica*, *Heterotrioza chenopodii*, *Bactericera nigricornis* and *Trioza urticae*. This report highlights the need for further investigation into the spread dynamics of the bacterium in other areas, to mitigate potential risks to agriculture.

## 1. Introduction

‘*Candidatus* Liberibacter solanacearum’ (‘Ca. L. solanacearum’ or CLso) is a notable plant pathogen affecting plants in the Solanaceae family, with its initial distribution and impact observed in North America and New Zealand. CLso is primarily associated with the “zebra chip” disease in potatoes (*Solanum tuberosum* L.) [1]. This Gram-negative bacterium interferes with nutrient transport by blocking the phloem, leading to various disease symptoms. Psyllids, insects of the superfamily Psylloidea, are the primary vectors of CLso [2,3], although the bacterium can also spread through infected plant material, such as potato tubers [4]. Due to its polyphagous nature, CLso represents a significant economic threat to numerous host plants across the Americas, New Zealand, and Europe [5].

In the Apiaceae family, CLso causes a range of symptoms that vary in appearance and severity depending on the host species and bacterial haplotypes. In carrots (*Daucus carota* L.), symptoms include leaf yellowing, purpling, curling, stunted growth, shoot abnormalities such as proliferation, and root alterations including the development of hairy secondary roots [6,7,8]. These symptoms collectively lead to systemic plant decline and reduced crop yields [9]. Parsley (*Petroselinum crispum* (Miller) Fuss) also exhibits CLso-related symptoms, most notably leaf yellowing [10]. However, these symptoms frequently overlap with those caused by other phytopathogens such as phytoplasmas and spiroplasmas, making accurate visual diagnosis challenging [10].

Numerous haplotypes of CLso have been reported, each exhibiting unique geographical distribution, as well as variations in their vector feeding behavior and the bacterium’s geographic spread [11]. Molecular tools, particularly sequencing and the comparison of single-nucleotide polymorphisms (SNPs) within specific genetic regions, are commonly used for haplotype differentiation. Key genetic regions include the 16S rRNA gene, the 16S-23S intergenic spacer region (ISR), and the 50S ribosomal protein genes [12,13].

Early studies identified CLso haplotypes A and B as the primary agents of diseases in the Solanaceae family, including potato zebra chip. Haplotype A was first reported in Central America and New Zealand [12] and both haplotypes A and B were subsequently confirmed in the USA and Mexico [14,15].

Haplotypes C, D, and E of CLso are primarily associated with plants in the Apiaceae family [16,17,18,19]. Haplotype C is frequently reported in carrots in European countries and has been linked to significant yield losses in some cases [20]. In Finland, haplotype C was associated with the psyllid *Trioza apicalis* Förster and severe carrot yield losses [21]. Additionally, this haplotype has been reported to cause mild leaf discoloration in parsley [17]. In Spain, haplotype C is associated with *Bactericera trigonica* Hodkinson (Hemiptera: Triozidae) [8]. Historical seed collections have revealed the presence of this haplotype in old celery seeds (Alba GS variety) collected in Germany in 1997 and parsley seeds (Bravour and Curlina varieties) from the UK between 1990 and 2005 [22]. Interestingly, haplotype C has been found to infect Solanaceae family plants near infected Apiaceae plants, including black nightshade (*Solanum nigrum* L.) [17], as well as potato plants located on the periphery of infected carrot fields, although these potato plants did not exhibit any characteristic zebra chip symptoms [23].

Haplotypes D and E were first identified in Scotland in parsley seeds [24]. Both haplotypes were confirmed to be strongly associated with the psyllid vector *B. trigonica*, as demonstrated in transmission trials involving carrot and celery plants (Teresani, et al. [25]. More recently, these haplotypes were found in association with *B. trigonica* in carrot fields in Italy [26]. In Italy, haplotype E was also detected in the seeds of two commercial carrot varieties, Berlicum and Mezza Lunga Nantese [27]. This haplotype has shown notable plasticity, being transmitted by *Bactericera nigricornis* Förster (Hemiptera: Triozidae), and it is capable of infecting both carrots and potatoes [28]. Additionally, it has also been observed in various Apiaceae vegetables, including celery, parsley, and chervil [16].

Haplotype U, so named for its detection in the common nettle (*Urtica dioica* L.), was first identified in Finland [17] and later reported in the UK. It is closely associated with the psyllid species *Trioza urticae* L. (Hemiptera: Triozidae) [11].

Haplotypes F and G have only been identified in the USA. Haplotype F was initially discovered in Oregon in potato tubers exhibiting zebra chip symptoms, such as streaking and dark medullary rays [29]. Haplotype G was later recorded in Southern California in *Solanum umbelliferum* Eschscholtz, a perennial native Solanaceae plant that serves as a natural host for the psyllid vector *Bactericera cockerelli* Šulc (Hemiptera: Triozidae), the principal vector of haplotypes A and B [30].

Haplotype H was first detected in Finland in 2018 in carrot and parsnip, exhibiting symptoms similar to those associated with haplotype C. It was also found in wild weeds, including wild black bindweed (*Fallopia convolvulus* (L.) Á.Löve) and pale smartweed (*Persicaria lapathifolia* (L.) Delarbre) [18].

Earlier, a related strain was reported in the USA infecting field bindweed (*Convolvulus arvensis* L.) and sweet potato (*Ipomoea batatas* (L.) Lam.) (Torres, et al. [31]. This strain was later classified as haplotype H through 16S rRNA gene analysis [32]. To differentiate the US strain, Sumner-Kalkun, Highet, Arnsdorf, Back, Carnegie, Madden, Carboni, Billaud, Lawrence and Kenyon [11] proposed the designation “haplotype H(Con)” due to its association with the Convolvulaceae family.

Haplotypes Cras1 and Cras2 are named in reference to the genus *Craspedolepta* (Hemiptera: Aphalaridae), as they were first identified in Scotland within two psyllid species belonging to this genus: *Craspedolepta nebulosa* Zetterstedt (Hemiptera: Aphalaridae) and *Craspedolepta subpunctata* Förster (Hemiptera: Aphalaridae) [11].

Haplotypes Aph1, Aph2 and Aph3 are associated with psyllids of the *Aphalara* genus and have been identified exclusively in the US [33]. Aph1 was found in *Aphalara loca* Caldwell (Hemiptera: Aphalaridae), while Aph2 (including variants Aph2a and Aph2b) was detected in *Aphalara persicaria* Caldwell (Hemiptera: Aphalaridae). All three haplotypes were found in *Aphalara curta* Caldwell (Hemiptera: Aphalaridae).

The psyllid species *B. trigonica* has been identified as an effective indicator of CLso distribution in Spain (including the Canary Islands) [8], as well as in France [16]. Its presence on CLso-infected Apiaceae plants has also been reported in Serbia [34]. A recent study in Italy reported a high prevalence of CLso within *B. trigonica* in the Abruzzo region, with 27.5% of tested psyllids carrying the bacterium [26].

The strong association of *B. trigonica* with Apiaceae plants, particularly carrots, has been confirmed by Ben Othmen, et al. [35]. While earlier studies suggested limited vectoring capabilities for *B. nigricornis* and *Bactericera tremblayi* Wagner (Hemiptera: Triozidae) [36,37], recent findings by Antolínez, Moreno, Ontiveros, Pla, Plaza, Sanjuan, Palomo, Sjölund, Sumner-Kalkun and Arnsdorf [28] demonstrated that *B. nigricornis* can transmit the bacterium to carrot and potato, underscoring the need for further field research. Additionally, unidentified *Bactericera* species were found positive for CLso in Tenerife [36].

Beyond *B. trigonica*, other psyllid species have been identified as hosts of CLso. For instance, *T. urticae*, which is associated with plants in the Urticaceae family, was first reported as a CLso host in Finland [17], with subsequent detections in Germany and Estonia [38,39]. This highlights the potential role of weeds, such as nettles, as reservoirs for the pathogen. Similarly, *Trioza anthrisci* Burckhardt (Hemiptera: Triozidae) has been confirmed as a carrier in Finland [23], Sweden, and Scotland [40] However it is not currently considered a significant threat to potato crops due to its early emergence [23]. In Finland, *T. anthrisci* typically emerges early in the spring, with peak egg-laying activity occurring in late May to early June, before potato sprouts emerge, thus minimizing the risk of pathogen transmission to potato plants.

‘Ca. L. solanacearum’ represents a significant phytosanitary threat to the production of various crops, particularly those in the Apiaceae family, across Europe. Since its initial detection in carrot crops in Finland and Spain in 2008, the bacterium has rapidly spread to other countries, infecting a broader range of host plants and causing substantial economic losses [20]. This situation highlights the potential role of multiple psyllid vectors in the transmission of this pathogen. It also underscores the urgent need for further research to better understand the dynamics of its spread and the risks of its establishment under field conditions in European agriculture. The present study aims to assess the current status of CLso in Northwestern Italy by surveying its known and potential host plants, while also investigating the dynamics of its potential psyllid vectors.

## 2. Materials and Methods

### 2.1. Sampling Sites, Dates, and Collection Methods of Psyllids and Plants

Field sampling was conducted in 2022 and 2023 across various locations in the Piedmont region, Northwestern Italy, involving both cultivated crops and wild plants, as detailed in Table 1.

The cultivated crops included celery (grown under tunnels), carrots (in open fields), and potatoes (in open fields). As for wild plants, the survey considered those growing within or around crop fields, as well as in uncultivated areas. In 2022, one or two sampling sessions were performed at each site using a sweep net and the beat tray method. In 2023, however, the sampling efforts were more systematic, with four sessions conducted on potatoes and eight to ten on carrots, complemented by using yellow sticky traps (25 cm × 20 cm, BIOGARD^®^ GLUTOR giallo, Biogard, Grassobbio, Italy).

All potato fields were sampled at two-week intervals during the last two months of the growing season to ensure that the plants were sufficiently developed to support psyllid populations while remaining resilient enough to withstand the sampling process. Specifically, two potato fields in Magnano (Magnano 1 and Magnano 2, approximately 0.2 hectares each) were sampled from late May to mid-July. Another potato field in Pontecurone (3.5 hectares) was sampled from early June to late July, while a smaller field in Avigliana (0.1 hectares) was monitored from late June until early August. Sampling activities in potato fields concluded just before harvest.

Regarding carrot fields, Dalmazzo 1 (0.6 hectare) was sampled at roughly two-week intervals from early June to late September, totaling eight visits. In the second carrot field, Dalmazzo 2 (0.9 hectare) the carrots were sown later, to ensure continuity in production. It was sampled every 7 to 10 days from late September to early December, totaling ten visits. This intensified sampling aimed to maximize psyllid collection during the onset of colder temperatures in early October, when populations typically decline. Additionally, Dalmazzo 2 was notable for its high weed diversity, which was hypothesized to support a broader range of psyllid species, making it a key site for investigation.

Each potato and carrot field was equipped with five traps: four at the corners of the field and one at its center. However, one potato field, “Magnano 1”, deviated from this pattern, utilizing only three traps that were arranged diagonally, with two at opposite corners and one in the center.

Psyllids captured on the sticky traps were detached by applying a drop of xylene to dissolve the glue, allowing for the careful removal of insects with minimal or no damage. The collected psyllids were then rinsed twice in pure ethanol to ensure proper preservation and cleanliness.

All adult psyllids were identified to the species level and sexed based on their morphological characteristics, following the taxonomic references of Hodkinson and White [41] and Ossiannilsson [42]. Both freshly collected psyllids and those captured on sticky traps were preserved in pure ethanol at −20 °C until DNA extraction. Priority was given to psyllids from sticky traps to minimize DNA degradation and loss.

Plants were identified based on Pignatti [43] and samples were collected from both wild and cultivated species, including both asymptomatic and symptomatic plants. Samples of leaves, leaves and stems, roots and veins were collected for each plant species and stored at −20 °C before DNA extraction.

### 2.2. CLso Detection

Psyllid DNA was isolated using two different protocols. Freshly collected insects (obtained via the beat tray and sweep net methods) were processed following an extraction protocol based on 10% Chelex (Merck, Darmstadt, Germany), as described by Casquet, et al. [44]. Briefly, the psyllids were dried from alcohol on filter paper and then placed into 1.5 mL microcentrifuge tubes. To each tube, 50 µL of 10% Chelex was added, followed by grinding with polypropylene micropestles. After thoroughly grinding the psyllids, 10 µL of Proteinase K (10 mg/mL) (Merck, Darmstadt, Germany) was added. The samples were then incubated at 55 °C for 24 h. Then, the samples were heated at 95 °C for 5 min, centrifuged at 10,000 rpm for 1 min, and stored at −20 °C, as outlined by Quintana, et al. [45].

Conversely, psyllids captured with sticky traps were processed following the protocol by Kannan, et al. [46] modified for insects. Individual psyllids were dried from alcohol and placed into a 1.5 mL tube, to which 300 µL of GBL extraction buffer was added along with 17 µL of Lysozyme 10 mg/mL (Merck, Darmstadt, Germany) and 3 µL of Proteinase K 50 µg/mL. Psyllids were then pestled in within the reagent mixture and incubated at 37 °C for 20 min. Immediately afterward, 150 µL NaCl (6M) (Merck, Darmstadt, Germany) and 200 µL chloroform (VWR International Srl, Milan, Italy) were added. The samples were then vortexed and centrifuged at 13,000 rpm for 10 min. The upper aqueous phase was then transferred to a new tube, and the DNA was precipitated by adding 0.6 volumes of isopropanol and incubating for one hour at −4 °C, followed by cold centrifugation at 13,000 rpm for 10 min and the removal of the supernatant. The DNA pellet was then washed with 500 µL of 70% ethanol and subjected to another cold centrifugation at 13,000 rpm for 5 min. Finally, the supernatant was discarded, and the pellet was suspended in 20 µL of TE 1× buffer.

Plant DNA was extracted from 1–2 g of vegetal tissues according to the EPPO [47] protocol. For green plant tissues, only the leaf veins were used, while, for carrot roots, a thin layer of root tissue along with secondary hairy roots was collected. Homogenization was performed in a Bioreba^®^ universal extraction bag (12 × 15 cm, Bioreba AG, Reinach, Switzerland) using the suggested extraction buffer (here referred to as PT extraction buffer) at a 1:2 (*w*/*v*) ratio. The material was manually ground with a porcelain pestle. Next, 300 µL of the homogenate was transferred into a 2 mL microcentrifuge tube to which 80 µL of Lysozyme (50 mg/mL) was added. The samples were vortexed and incubated at 37 °C for 30 min. After incubation, CTAB buffer was added to the homogenate, and the mixture was incubated at 65 °C for 30 min. After 3 min at room temperature, 500 µL of ice-cold chloroform was added. The mixture was vortexed and centrifuged at 13,000 rpm for 10 min. The upper aqueous phase was transferred to a new 1.5 mL microcentrifuge tube, and the DNA was precipitated and washed using the same procedure described for insect DNA extraction. However, the DNA pellet from plant samples was resuspended in 100 µL of TE 1× buffer. DNA concentration and quality were assessed using a NanoDrop^®^ ND-1000 spectrophotometer (NanoDrop Technologies, Inc., Wilmington, DE, USA).

‘*Candidatus* Liberibacter solanacearum’ (CLso) detection was performed via conventional PCR using the specific primers Lso TX 16/23 F (5′-AATTTTAGCAAGTTCTAAGGG-3′) and Lso TX 16/23 R (5′-GGTACCTCCCATATCGC-3′), which target a conserved region within the 16S-23S intergenic spacer region (ISR) [48] following the EPPO [47] guidelines. The optimized PCR reaction mixture, based on the protocol suggested by Ravindran, Levy, Pierson and Gross [48], contained: 28.7 μL of ddH_2_O, 5 μL of 10× PCR buffer, 4 μL of MgCl_2_ (25 mmol/L), 5 μL of dNTPs (2 mmol/L), 2.5 μL of each primer (10 μmol/L), 0.3 μL of Taq polymerase (2.5 U/μL), and 2 μL of DNA template, for a total reaction volume of 50 μL.

The PCR cycling conditions based on Ravindran, Levy, Pierson and Gross [48] were modified as follows: initial denaturation at 95 °C for 15 min; 35 cycles of denaturation at 95 °C for 30 s, annealing at 55 °C for 45 s, extension at 72 °C for 95 s, and final extension at 72 °C for 10 min. Samples were stored at 4 °C until further analysis.

Amplified DNA was subjected to electrophoresis at 70 V for approximately 95 min. The gel was then stained with a 30% GelRed solution (Merck, Darmstadt, Germany) for 25 min with gentle shaking, and DNA bands were visualized using a Gel Doc™ EZ Imager (Bio-Rad Laboratories Inc., Hercules, CA, USA).

### 2.3. CLso Characterization

CLso DNA from positive samples was subjected to conventional Sanger sequencing following amplicon purification with the QIAquick^®^ PCR Purification Kit (QIAGEN, Hilden, Germany), following the manufacturer’s protocol. To ensure sequence quality and accuracy, chromatograms were visually inspected using Chromas v2.6.6 software (Technelysium Pty. Ltd., Brisbane, Australia). Reverse complement generation, sequence alignment, and consensus sequence construction were performed using BioEdit v7.2.5 software [49]. The consensus sequences were compared to reference sequences in the NCBI (National Center for Biotechnology Information) GenBank database using BLAST v2.16.0+ (NCBI, Bethesda, MD, USA) to confirm species identification. Sequences with an E-value of 0 and >97.5% identity were selected, excluding complete genomes [50].

Recognizing that some isolates were represented by multiple identical sequences, a single representative sequence from each isolate group identified in the reports was selected for the phylogenetic analysis to improve clarity and avoid redundancy. Moreover, two additional sequences (KU041863.1 and KT984852.1), derived from 16/23S rDNA extracted from commercial carrot seeds sourced from Italy [27], were incorporated into the phylogenetic tree. These sequences were included for their relevance in terms of the geographical source, enhancing the taxonomic resolution and relatedness insights provided by the tree. A single representative sequence of *Candidatus* Liberibacter asiaticus (Las) was included in the construction of the phylogenetic tree as an outgroup on which the constructed tree was rerooted.

Multiple sequence alignment was performed using ClustalW in BioEdit v7.2.5, and a phylogenetic tree was constructed with MEGA v11.0.13 software [51,52]. Subsequently, the best-fit DNA/protein substitution model for phylogenetic tree construction was determined using the maximum likelihood (ML) approach, also available in MEGA v11.0.13. Phylogenetic tree reconstruction was then conducted using the selected substitution model and the maximum likelihood method via bootstrap analysis with 1000 replicates and partial deletion, retaining sites with at least 95% coverage. The produced phylogenetic tree was then handled for better visualization using the online tool iTOL v6 (EMBL, Heidelberg, Germany) [53].

## 3. Results

### 3.1. Psyllid Survey

Two psyllid species were predominantly collected on wild plants: *Heterotrioza chenopodii* Reuter (Hemiptera: Triozidae) from *Chenopodium album* L. and *T. urticae* from *U. dioica* and *Parietaria officinalis* L. Additionally, *Trioza alacris* Flor (Hemiptera: Triozidae) adults were collected in August from *Laurus nobilis* L. in Rivalta di Torino (TO). Fig psyllid adults (*Homotoma ficus* L.) (Hemiptera: Carsidaridae) were collected from *Ficus carica* L. at three distinct locations: Rivalta di Torino (TO), Grugliasco (TO), and Benna (BI).

Manual collection techniques, such as the beat tray and sweep net methods, proved ineffective in capturing psyllids across all four potato fields throughout the season. This was the case for both commercial potato plants and surrounding weed species. Similarly, no psyllids were collected from tomato fields in Pontecurone (AL) during the July sampling activity. Conversely, sticky traps deployed within these fields yielded positive results in three out of the four regularly monitored locations by capturing adult psyllids. However, the Pontecurone (AL) potato field remained apparently devoid of psyllids, as confirmed by the absence of captures on sticky traps during all monitoring visits.

In contrast, manual collection techniques were highly effective in gathering a substantial number of adult psyllids from the carrot field “Dalmazzo 1”, with a total of over 1140 individuals, whereas “Dalmazzo 2” resulted in the capture of only 3 adult psyllids. Sticky traps were highly efficient in capturing psyllids from both carrot fields.

Across all potato fields, *B. nigricornis* was the dominant species, followed by *H. chenopodii*; meanwhile, in carrot fields, *B. trigonica* was the most abundant species, followed by *H. chenopodii*. Interestingly, a small number of *Aphalara freji* Burckhardt and Lauterer (Hemiptera: Aphalaridae) individuals were also detected on sticky traps in “Magnano 2”. A summary of the psyllids collected during the surveys for CLso across all locations is presented in Table 2. Additionally, the numbers of collected psyllids by the sticky traps in each time point are available in Tables S1–S3 of the Supplementary Material.

**Figure 1:**
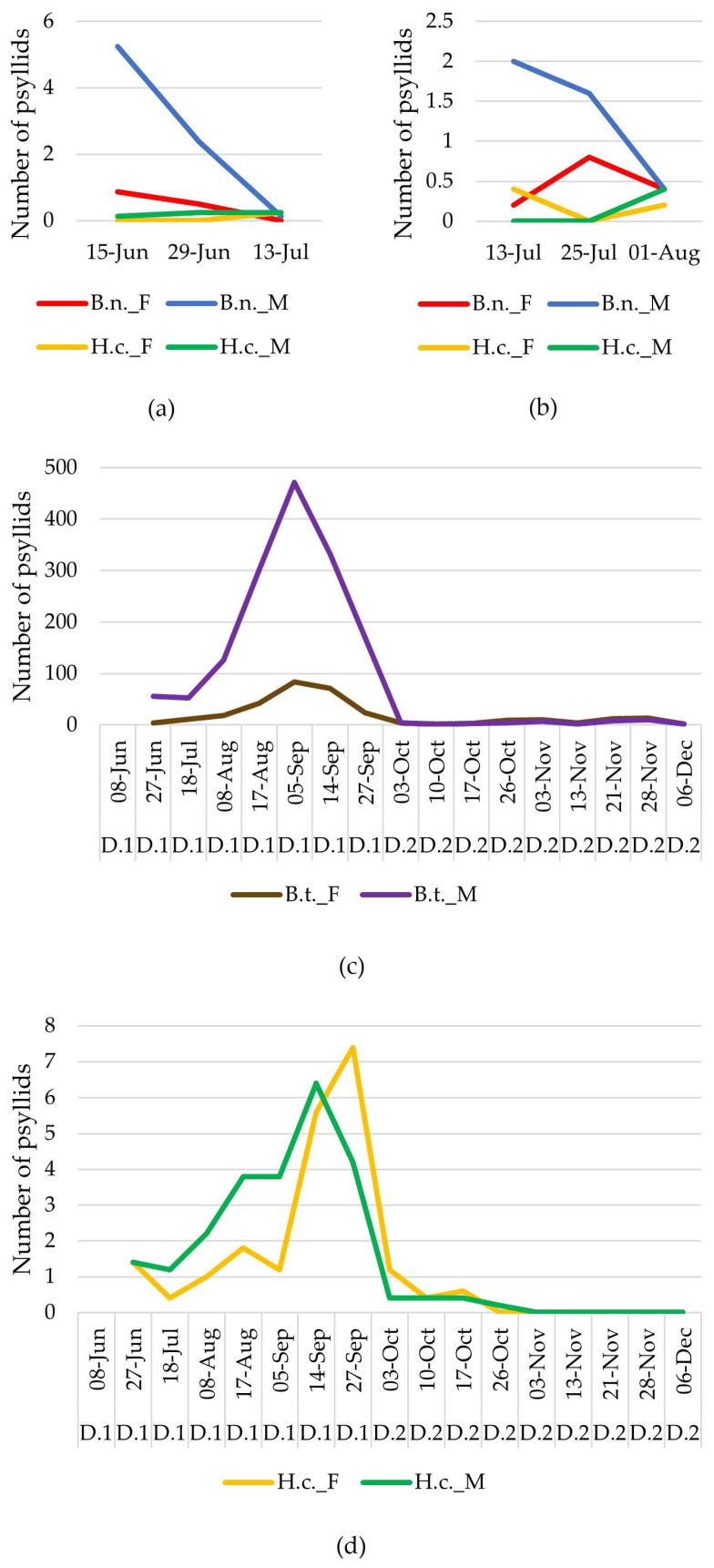
Average number of psyllid adults collected using sticky traps in 2023: (**a**) *Bactericera nigricornis* (B.n.) and *Heterotrioza chenopodii* (H.c.) in Magnano potatoes fields; (**b**) *Bactericera nigricornis* (B.n.) and *Heterotrioza chenopodii* (H.c.) in Avigliana potatoes field; (**c**) *Bactericera trigonica* (B.t.) in both carrot fields; (**d**) *Heterotrioza chenopodii* (H.c.) in both carrot fields, Locations: (D.1 = Dalmazzo 1, D.2 = Dalmazzo 2), Sex (F = female, M = male).

The average of the psyllid catches within the traps of each field, categorized by species and sex, was used to draw the graphs in Figure 1. Given their proximity and similar environmental conditions, the two Magnano fields were combined for this analysis. Notably, male *B. nigricornis* consistently outnumbered females, except for the final sampling date on 1 August, when the sex ratio was equal. The number of captured males of *B. nigricornis* from “Magnano 2” on 15 June was significantly high, due to overall high trap catches, as well as two specific traps recording 9 and 22 males.

In the two carrots fields of Dalmazzo, the visits to “Dalmazzo 2” were conducted immediately after completing the visits to “Dalmazzo 1”, as shown in Figure 1, providing a longer observation window for psyllid species presence. A relatively stable sex ratio was observed among the manually collected *B. trigonica* individuals. However, a marked disparity emerged in trap captures from the “Dalmazzo 1” field, where males significantly outnumbered females from June to mid-September. Interestingly, this trend was reversed in the “Dalmazzo 2” field, where the initial sex ratio was balanced, followed by slight female dominance from mid-October to late November.

Peak abundances of *H. chenopodii* were observed in both manual collections and sticky trap captures around mid- to late September. A significant decline in *H. chenopodii* populations was noted in the “Dalmazzo 2” carrot field by early October. While low numbers persisted until mid-October, the species was absent from both the manual and trap collections thereafter. No discernible sex-specific patterns were observed in *H. chenopodii* populations throughout the study period.

### 3.2. CLso Detection

The PCR analyses revealed a low level of CLso infection across various psyllid species and locations sampled in 2022 (Table 3). Among the species examined, *H. chenopodii*, which was highly abundant in different semi-wild niches in the Biella, Cuneo, and Torino provinces, raised concerns due to its potential as a vector. However, no positive individuals were found, suggesting low susceptibility or vector competence for this bacterium. In contrast, *T. urticae* exhibited a higher infection rate, particularly among females. Notably, one female tested positive in Benna (BI) on *U. dioica*, and one female out of out of four psyllids tested positive in S. Ambrogio (TO) on the non-native host plant *P. officinalis*. CLso was not detected in the samples collected from *H. ficus* and *T. alacris* (Table 3). Additionally, none of the *S. physalifolium* plant samples collected in 2022 tested positive for CLso.

The CLso PCR detection tests conducted in 2023 revealed different levels of infection within various psyllid species and host plants. The majorly abundant psyllid species within potato fields, *B. nigricornis*, was found to be positive for CLso, confirming its status as a potential vector for the bacterium in the territory. However, the infection rate was very low, with only one male individual testing positive; this specimen was collected from the “Magnano 2” field. In contrast, no psyllids were found to be positive among *H. chenopodii* and *A. freji* individuals collected from potato fields.

In carrot fields, positive samples for CLso were found among individuals of both psyllid species, including *H. chenopodii,* which had not been found to host the bacterium in potato fields. In “Dalmazzo 1”, a relatively high prevalence of infection was observed in *B. trigonica*, with 6 out of 156 females and 3 out of 124 males testing positive. Despite the high abundance in this field of *H. chenopodii* and its primary host, *C. album*, no individuals of this species, whether collected from carrot plants or directly from *C. album*, were found to be infected with CLso. *Bactericera trigonica* collected from the “Dalmazzo 2” carrot field showed a lower prevalence of CLso, with 2 out of 139 males testing positive. Moreover, unlike in “Dalmazzo 1”, *H. chenopodii* had an infection with CLso, with 2 out of 11 females and 1 out of 6 males testing positive. These results indicate that the prevalence of the bacterium varies significantly among psyllid species and host plants, suggesting potential differences in vector competence and transmission efficiency (Table 4).

The conventional PCR analysis conducted on the DNA samples extracted from different plant species and locations revealed the limited presence of CLso. Among the numerous samples tested across various plant parts, only a small subset exhibited positive results. Specifically, the only positive samples were detected in carrot plants from the “Dalmazzo 2” location, where positive results were found from both psyllids species *B. trigonica* and *H. chenopodii*. Notably, these positive samples were exclusively found in the veins of the plants, with 3 out of 39 samples yielding positive PCR results. In contrast, in the “Dalmazzo 1” field, which had a slightly higher number of positive-CLso insects, no positive plant samples for CLso were found. Moreover, none of the plant samples collected from potato plants or weed plants from the Avigliana and Pontecurone fields tested positive for CLso (Table 5).

**Figure 2:**
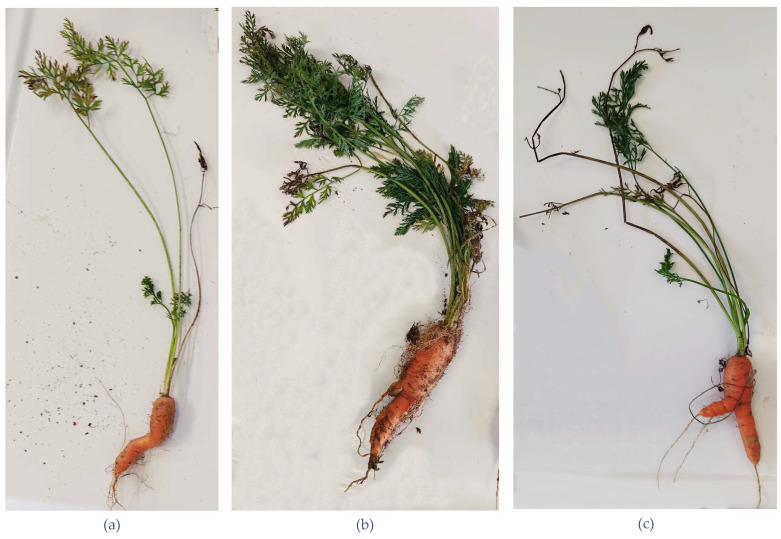
Observed symptoms on carrot plants: (**a**) leaf discoloration and curling; (**b**) root abnormalities and hair-like secondary roots; (**c**) stunted growth and root alteration.

In the Pontecurone potato field, several plants exhibited classic symptoms of CLso infection, including leaf yellowing, upward leaf curling, shortened internodes, and premature wilting. However, although samples were collected from symptomatic plants, none tested positive for CLso. In both carrot fields, numerous plants displayed characteristic CLso symptoms, such as leaf discoloration and reddening, leaf curling, stunted growth, and root abnormalities (Figure 2). A significant number of these symptomatic samples were included in the survey, with 66 from “Dalmazzo 1” and 14 from “Dalmazzo 2” undergoing testing. Interestingly, of the three positive plant samples identified in this study, only two exhibited visible symptoms, while the remaining positive sample was derived from an asymptomatic plant with normal green foliage (Table 6).

### 3.3. CLso Characterization

Sequences of ‘*Candidatus* Liberibacter solanacearum’ detected in *B. nigricornis* collected from traps in potatoes field and *T. urticae* collected from *U. dioica* and from *P. officinalis* ranged from 344 to 392 bp in length. These resulting sequences all had almost the same hits on NCBI database. One CLso sequence from *B. nigricornis* and two sequences of CLso from *T. urticae* collected from *U. dioica* and from *P. officinalis* were deposited in GenBank under the accession numbers PV394705, PV397476, PV397481, respectively. Similarity of 100% and an E value of 0 were observed with isolates of CLso haplotype U from *T. urticae* in Scotland, United Kingdom, under the accession numbers MT230511.1, MT230513.1, MT230517.1, MT230522.1, MT230525.1, MT230528.1 and MT230529.1.

The positive control for CLso used in this study, isolated from carrot seeds in Italy, as well as the CLso-positive sample from carrot plant leaf veins, shared nearly identical sequencing results with those of the two psyllid species *B. trigonica* and *H. chenopodii.* A representative sequence of the bacterial clone used as a positive control (from carrot seeds) and the sequence detected in leaves of *D. carota*, *B. trigonica* and *H. chenopodii* were deposited in GenBank under accession numbers PV397480, PV397479, PV397478, and PV397477, respectively. All these sequences were around 390 bp in length and showed hits on the NCBI databases with up to 21 isolates, demonstrating a 0 E value and identity scores above 97.92%. Three isolates exhibited 100% identity and a 0 E value, including CLso haplotype D, isolated from historical celeriac seeds (accession number KY619977.1), as well as isolates from carrot leaves in Greece (KY595982.1 and KY595983.1).

A complete genome sequence of the zebra chip strain ‘*Candidatus* Liberibacter solanacearum’ (accession number CP002371.1), isolated from *B. cockerelli* in Texas, USA, showed consistent E values of 0 and identity scores above 98% in BLAST searches against all query sequences from both groups. However, due to the significantly larger genome size (1,258,278 bp), this reference sequence was excluded from the subsequent phylogenetic analysis to avoid potential bias when compared to the shorter Sanger sequencing reads.

**Figure 3:**
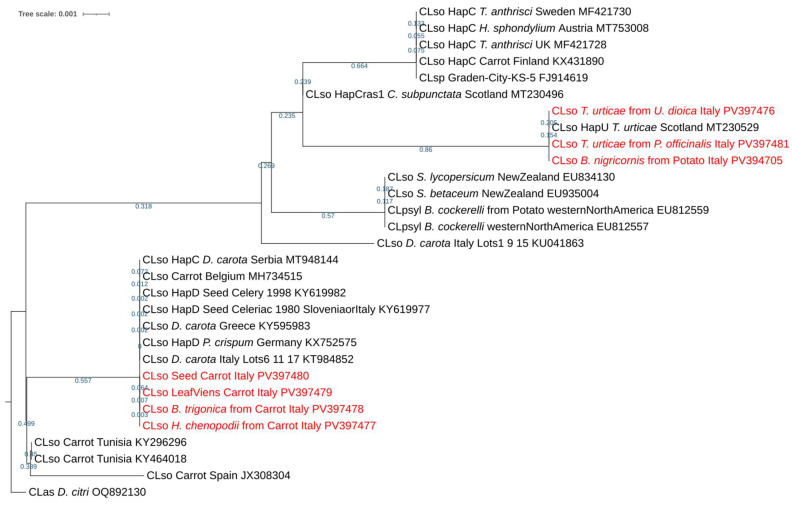
Phylogenetic tree of the CLso query of each species with the top hits from the BLAST database after removing repetition and considering the addition of the two sequences from commercial carrots seeds in Italy.

The phylogenetic tree presented in Figure 3, constructed using the Kimura 2-parameter model, depicts the relatedness among ‘*Candidatus* Liberibacter solanacearum’ isolates. The tree was built based on maximum likelihood from the alignment of 16-23S rRNA sequences, and it was rooted with the outgroup sequence ‘*Candidatus* Liberibacter asiaticus’, which is a closely related bacterium species.

The tree clearly shows that the study’s query sequences form two distinct groups. CLso isolated from *B. nigricornis* and *T. urticae* clustered with isolates related to the CLso haplotype U, while CLso detected in *D. carota*, *B. trigonica* and *H. chenopodii* appeared to be more closely related to haplotype D.

An interesting finding was the presence of CLso haplotype U, previously reported in *T. urticae*, in *B. nigricornis*, suggesting a broader host range for this bacterium than previously recognized. Additionally, sequences isolated from various species within the carrot fields clustered into a clade with isolates that were all from the Apiaceae family, including carrot, celery, and parsley. This clustering highlights a kind of common geographical distribution, including Germany, Greece, Slovenia, Italy, Serbia, and Belgium. Notably, CLso isolates from carrots in Finland grouped together with isolates of *T. anthrisci* from the UK, further emphasizing the shared geographical and ecological associations. These observations suggest not only the diversity and broad host range of CLso but also the complex geographical and ecological patterns of its distribution across different plant hosts and psyllid species.

The sequences obtained from the carrot field samples in this study aligned perfectly with the reference sequence KT984852.1, which was derived from commercial carrot seeds in Italy [27]. This alignment confirmed that our sequences are part of the “second group of isolates” characterized by haplotypes C or D in the original study conducted by Ilardi et al. (2016). In contrast, the sequence KU041863.1, which was also derived from the same study, came from a different leaf and clustered separately in the phylogenetic tree (Figure 3).

## 4. Discussion

Although the presence and spread of ‘*Candidatus* Liberibacter solanacearum’ in Europe raise some concern, reports of its presence in Italy have been rather sporadic. The first detection was reported in 2016 in carrot seeds [27], followed by its identification in a few carrot plants in Sicily a year later [54]. In 2024, the presence of the bacterium in carrot plants from the Abruzzo region (Central Italy) was reported [55]. That same year, it was reported that haplotypes D and E had been detected in *B. trigonica* individuals collected from carrot fields in the same region [26].

In this context, this is the first report of CLso in carrot plants, as well as in the associated psyllid *B. trigonica,* both carrying haplotype D, in Northwestern Italy. Moreover, it highlights the presence of CLso in the occasional psyllid *H. chenopodii*, which had already been reported by Grimm, Horton, Lewis, Garczynski, Jensen and Charlton [33] in the United States as being capable of hosting a new haplotype of the bacterium. In addition to haplotype D, the present study also allowed us to detect haplotype U, in the psyllid *T. urticae*, collected on the wild plants *U. dioica* and *P. officinalis*.

Similarly, this study represents the first report, in Italy, of CLso in the generalist psyllid *B. nigricornis*, collected in a potato field, as well as the association of this insect with haplotype U. This scenario provides new and interesting insights for the study of the spread and epidemiology of CLso in Italy.

Wild plants have been confirmed as a concerning source of bacterial inoculum, as well as a reservoir for potential psyllid vectors. CLso was detected in two female specimens of the psyllid species *T. urticae*, which had previously been identified as a host of the bacterium in Finland, Germany, and Scotland [11,17,38]. These insects were collected from two different host plant species within the Urticaceae family: *P. officinalis* and *U. dioica*, which are known to be the principal hosts for this oligophagous psyllid [56]. The latter was previously found to be positive for CLso in Finland and Scotland [11,17]. The sites in which these two specimens were collected, located in the provinces of Turin and Cuneo, are considerably far from each other, suggesting the broad geographical distribution of the bacterium among these insects and their host plants within the study area. However, the detection of CLso in only two individuals indicates the low presence of the bacterium; this is consistent with findings in Germany, where Sjolund, Arnsdorf, Carnegie, Fornefeld and Will [38] detected CLso in just a single *T. urticae* specimen.

*Heterotrioza chenopodii* is an oligophagous, generally bivoltine species that feeds on Amaranthaceae plants including *Beta vulgaris* L., *Convolvulus* spp.*, Atriplex* spp. It exhibits distinct seasonal patterns on *C. album,* with fluctuations driven by environmental conditions and host plant availability. A sharp decline in psyllid densities is typically observed during the hot summer and cold winter months [57], likely explaining the low numbers of *H. chenopodii* captured during the summer sampling of 2022. Nevertheless, it is noteworthy that, in our study, *H. chenopodii* was present in the sampled fields throughout the summer months, which contradicts the findings of Soliman, Malash and El-Hawagry [57], who reported the complete absence of this psyllid during the summer. This difference may be due to the fact that Soliman, Malash and El-Hawagry [57] conducted their study in Egypt, where significantly higher summer temperatures may limit psyllid activity, compared to our study area in northern Italy. In our study, the presence of this insect, not only in uncultivated areas but also in carrot and potato fields, is undoubtedly linked to the presence of weeds within and around these fields. Weeds were particularly abundant, for example, in the potato fields of Avigliana and Magnano.

As for the *H. chenopodii* individuals directly collected from carrot plants, their presence can be considered occasional and sporadic, as is the case on *U. dioica*, since these species do not fall within the known range of host plants. However, this aspect warrants further investigation, as it could have significant implications for the epidemiology of CLso.

The total absence of psyllids from the potato field at Pontecurone is a fairly remarkable observation; it is likely due to the field’s well-maintained cleanliness, which prevents the presence of weeds that could serve as potential hosts. However, psyllids were not observed on the weed species growing in the adjacent areas nor in the nearby tomato field throughout the sampling period. This suggests that other factors may be at play, such as regular pesticide application; such factors known to play a significant role in suppressing psyllid populations in commercial fields [58].

The sporadic presence of *A. freji* in the potato field of Magnano 2 is undoubtedly due to the abundant presence of weed species, including members of the Polygonaceae family, which are the primary host of psyllids from the *Aphalara* genus [59,60].

While surveys of potato and carrot plants in Spain revealed the presence of *B. trigonica* and *B. nigricornis* on both plants, with the former being more abundant on carrots and the latter more common on potatoes [28,61], our findings present a more distinct and defined pattern: *B. trigonica* was captured solely on carrots, while *B. nigricornis* was found exclusively in potato fields. *Bactericera nigricornis*’s host range is exceptionally broad among psyllids, including cultivated and wild plants within the Solanaceae, Apiaceae, Amaranthaceae, and Brassicaceae families [62,63]. Between the two crops, however, potato seems to be the preferred host, as the nymphs are commonly found on this plant, while, in laboratory conditions, rearing immature stages of *B. trigonica* and *B. nigricornis* on potatoes resulted in the development of only *B. nigricornis* adults [61].

*Bactericera trigonica*, on the other hand, is known to prefer apiaceous plants [62], particularly carrots over celery, though they were grown in close proximity [36]. However, this psyllid is polyphagous, allowing it to overwinter and lay eggs on the buds of short shrubs that grow to less than 25 cm above the soil surface in fields [64]. This overwintering habit could explain the absence of psyllids in manual sampling of carrot plants carried out in late season, as the insects may have already moved to smaller plants within the fields.

An unbalanced sex ratio was observed in both *Bactericera* species with males being captured in significantly higher numbers than female, particularly during the summer months. This pattern is consistent with findings on the tomato potato psyllid (*B. cockerelli*) [65], moreover, the pear psyllid exhibits a significant male bias during reproductive periods, attributed to mate-seeking behavior [66]. Arismendi, et al. [67] reported a similar male bias in cicadellids, suggesting that both sexes are attracted to yellow sticky traps, but males may be more active, particularly during mating periods. The observed sex ratio bias in trap catch data, as opposed to manual collection data, suggests that this may not reflect true population differences but rather differences in movement behavior. It is plausible that males, particularly during mating periods, are more active and thus more likely to be captured on sticky traps. On the other hand, females may be less active, particularly during egg-laying periods, and are therefore less likely to be captured. This hypothesis aligns with the explanation proposed by Horton [66].

Both *B. trigonica* and *B. nigricornis* have been identified as vectors for CLso with the potential to transmit the bacterium to carrot and potato plants [28]. However, previous studies suggested that *B. nigricornis* might not be fully capable of vectoring CLso [36]. In Italy, *B. trigonica* collected from carrots was found to host CLso with a prevalence rate of 27.5% among the sampled psyllids [26]. While *B. trigonica* shows a strong preference for oviposition on Apiaceae plants [26], it is also highly efficient in transmitting the bacterium to potato plants [37]. These transmission dynamics highlight the risks of CLso spreading to new hosts that were not considered previously.

The old-world psyllid *H. chenopodii* has not been extensively surveyed for ‘Ca. Liberibacter’ species, despite sharing multiple host plants with other known CLso-vectoring psyllids. Cooper, et al. [68] included it as an outgroup in a research study of the bacterial symbiont of other psyllid species. In their study, individuals of *H. chenopodii* were tested for CLso, but the bacterium was not detected in any of the specimens. However, it was found in other psyllid species, including *B. cockerelli* and *Bactericera maculipennis* Crawford (Hemiptera: Triozidae). In contrast, another study that surveyed CLso included various psyllid species, and four specimens were found to host the bacterium. These were later identified molecularly as *H. chenopodii* collected from the Klamath Basin, USA [33].

A shift in the vector species of a ‘Ca. Liberibacter solanacearum’ haplotype could significantly increase the risk posed to a broader range of crop plants. This could lead to infections in host plants commonly used by multiple psyllid species. For instance, this could affect coniferous trees, such as *Picea abies* (L.) that act as overwintering shelters for *T. urticae* and other psyllid genera, including *Bactericera, Cacopsylla,* and *Trioza* [69]. A similar situation occurs with psyllids that vector fruit tree phytoplasmas and overwinter on conifers; however, to date, no conifers have been reported as positive for these pathogens [70,71]. Additionally, weed species acting as alternative hosts or feeding plants for psyllids have been largely under-researched in Europe to date.

The detection of CLso in carrot plant samples and its associated psyllid in Northwestern Italy, along with similar reports from Central Italy, raises concerns about the potential geographical spread of the bacterium [26,55]. In this study, no direct correlation was observed between the detection of CLso and the presence of specific symptoms on the tested samples, which is consistent with the findings of Tizzani, Bertinelli, Bertin and Ilardi [55], who concluded that there is no consistent correlation between CLso infection and symptom expression. It is also important to consider that symptoms of CLso can resemble those caused by other biotic and abiotic factors, leading to potential misdiagnosis [72,73].

Sequences from the isolates of this study clustered within a phylogenetic tree alongside ‘*Candidatus* Liberibacter solancearum’ sequences recorded in NCBI, distinct from other ‘*Candidatus* Liberibacter’ species. Within this cluster, our sequences were separated into two distinct groups: Group I, consisting of CLso isolates from *B. nigricornis* and *T. urticae*; and Group II, comprising CLso isolates from *D. carota*, *B. trigonica*, and *H. chenopodii*. Our sequences of *T. urticae* and *B. nigricornis* showed complete similarity with haplotype U of CLso initially reported in Finland on *T. urticae* and symptomatic *U. dioica* [17]. Moreover, it was subsequently reported on both hosts in Scotland, whose sequences showed complete similarity to ours [11]. Notably, this is the first reported detection of Haplotype U in *B. nigricornis*, highlighting the potential spread of the bacterium by new vectors, raising concerns about the risk of transmission to potatoes. It also prompts questions about how symptoms might manifest on potato plants. Furthermore, these observations suggest not only the diversity and broad host range of CLso but also the complex geographical and ecological patterns of its distribution across various plant hosts and psyllid species.

The second group of our sequences in the phylogenetic tree showed a close relationship with CLso isolates from apiaceous hosts, specifically from haplotypes D, along with other isolates whose haplotypes were not definitively identified, and one isolate belonging to haplotype C. This group primarily clustered with isolates from carrot plants in Italy, Greece, Belgium, and Serbia [27,34,74,75]. Interestingly, historical seed samples from celery in Serbia and parsley in Germany also clustered within the same branch [22,24]. Additionally, a closely related branch within the same clade included carrot isolates from Tunisia and Spain [35,76].

The phylogenetic tree reveals diversity within the CLso isolates found in carrot plants, indicating that different geographical locations or plant sources may harbour distinct CLso haplotypes. The clustering of these sequences further underscores the complexity of CLso’s distribution and suggest the potential existence of multiple haplotypes within carrot-associated populations across various regions. The close geographic distribution of most haplotype C, D, and U across Europe and the Mediterranean, coupled with the relatively short branch lengths in the phylogenetic tree, suggests a common evolutionary origin and the potential for easy dissemination among various psyllid vectors and plant hosts. The observed genetic variations within these haplotypes may affect their virulence on host plants. Notably, European haplotypes C, D, and U did not demonstrate the same level of aggressiveness as haplotypes A and B, which are associated with severe damage to potatoes in the USA and New Zealand [77,78].

Within haplotypes A and B, both of which exhibit varying degrees of aggressiveness on Solanaceae, notable differences exist. Haplotype B is associated with higher disease incidence and more severe symptoms in potato tubers compared to haplotype A, which generally induces milder symptoms [79]. A genomic analysis of the two most economically significant haplotypes of CLso, A and B, revealed distinct variability in unique coding sequences in A that were absent in B. The virulence of the haplotypes and their interactions with the host are likely attributed to this genetic variability [80]. When considering these findings in relation to the European haplotypes, which are currently regarded as less economically significant due to their relatively mild impact on crops, considerable concern arises about the potential for these haplotypes to evolve into more aggressive forms. Therefore, it is crucial to further monitor the presence, genetic diversity, and virulence of CLso populations in Europe to mitigate potential risks to agriculture.

## 5. Conclusions

This study reports the presence of CLso in Piedmont (Northwestern Italy), with evidence suggesting the occurrence of two haplotypes: haplotype D in carrot plants and its associated psyllid *B. trigonica*, as well as occasionally *H. chenopodii*, and haplotype U in the psyllids *B. nigricornis* and *T. urticae*. These findings strongly suggest the need for further surveys in other fields within the same region as well as other Italian regions. Additionally, investigating the transmission dynamics of different haplotypes via potential psyllid vectors within a risk assessment framework is highly recommended.

## Figures and Tables

**Table 1:** Psyllid sampling sites and collection details.

Year	Site	Host Plant	Sampling Method	Sampling Date/Period	Number of Visits
2022	Govone (CN) (44°47′40.3″ N 8°06′51.8″ E)	*Apium graveolens*	beat tray	31/05 07/06	2
	Grugliasco (TO) (45°04′12.8″ N 7°35′38.5″ E)	*Solanum nigrum* *Daucus carota* (wild)	beat tray	28/07	1
	Giaglione (TO) (45°08′35.6″ N 7°01′07.1″ E)	*Fraxinus* sp. *Urtica dioica* *Urtica urens*	beat tray sweep net	08/08	1
	Borgone Susa (TO) (45°07′09.7″ N 7°13′30.7″ E)	*Chenopodium album*	beat tray sweep net	08/08	1
	Chiusa di San Michele (TO) (45°06′02.2″ N 7°19′43.5″ E)	*Convolvulus arvensis*	beat tray sweep net	08/08	1
	Sant’Ambrogio di Torino (TO) (45°06′14.8″ N 7°20′49.1″ E)	*Urtica dioica* *Artemisia vulgaris* *Parietaria officinalis* *Chenopodium album*	beat tray sweep net	08/08 24/08	2
	Rivalta di Torino (TO) (45°01′40.9″ N 7°30′18.7″ E)	*Laurus nobilis* *Ficus carica*	beat tray	25/08	1
	Grugliasco (TO) (45°03′59.3″ N 7°35′32.5″ E)	*Ficus carica*	beat tray	07/09	1
	Benna (BI) (45°31′06.5″ N 8°07′16.8″ E)	*Ficus carica*	beat tray	02/10	1
	Lagnasco (CN) (44°37′26.3″ N 7°34′46.8″ E)	*Chenopodium album*	beat tray	01/10	1
	Benna (BI) (45°30′31.5″ N 8°07′54.1″ E)	*Urtica dioica* *Chenopodium album*	beat tray	02/10	1
2023	Magnano 1 (BI) (45°27′56.4″ N 7°59′50.7″ E)	*Solanum tuberosum*	beat tray sweep net sticky traps	30/05–13/07	4
	Magnano 2 (BI) (45°27′57.3″ N 7°59′49.5″ E)	*Solanum tuberosum*	beat tray sweep net sticky traps	30/05–13/07	4
	Pontecurone (AL) (44°56′00.7″ N 8°56′19.9″ E)	*Solanum tuberosum* *Convolvolus arvensis* *Solanum physalifolium* *Amaranthus* sp. *Daucus* sp. *Galinsoga* sp. *Heliotropium* sp.	beat tray sweep net sticky traps	08/06–27/07	4
	Avigliana (TO) (45°04′40.1″ N 7°24′24.5″ E)	*Solanum tuberosum* *Amaranthus retroflexus* *Convolvolus arvensis* *Galinsoga parviflora*	beat tray sweep net sticky traps	26/06–01/08	4
	Pontecurone (AL) (44°55′53.8″ N 8°56′06.6″ E)	*Solanum lycopersicum*	beat tray sweep net	27/07	1
	Dalmazzo 1 (CN) (44°21′11.2″ N 7°30′54.4″ E)	*Daucus carrota* *Chenopodium album* *Trifolium* sp.	beat tray sweep net sticky traps	08/06–27/09	8
	Dalmazzo 2 (CN) (44°21′13.2″ N 7°31′08.5″ E)	*Daucus carrota* *Amaranthus blitoides* *Amaranthus hybridus Solanum nigrum* *Senecio squalidus* *Galinsoga parviflora*	beat tray sweep net sticky traps	27/09–06/12	10

**Table 2:** Total male (M) and female (F) psyllids collected during the survey for CLso (BT = beat tray, SN = sweep net, ST = sticky trap).

Location	Method	*B. trigonica*		*B. nigricornis*		*H. chenopodii*		*T. urticae*		*T. alacris*		*A. freji*		*H. ficus*	
		F	M	F	M	F	M	F	M	F	M	F	M	F	M
Grugliasco	BT	0	0	0	0	0	0	0	0	0	0	0	0	0	3
Giaglione	BT/SN	0	0	0	0	0	0	0	1	0	0	0	0	0	0
B. Susa	BT/SN	0	0	0	0	2	1	0	0	0	0	0	0	0	0
S. Ambrogio	BT/SN	0	0	0	0	22	13	8	7	0	0	0	0	0	0
Rivalta	BT	0	0	0	0	0	0	0	0	1	3	0	0	1	3
Benna	BT	0	0	0	0	43	42	1	0	0	0	0	0	23	18
Lagnasco	BT	0	0	0	0	18	8	0	0	0	0	0	0	0	0
Magnano 1	ST	0	0	5	8	0	2	0	0	0	0	0	0	0	0
Magnano 2	ST	0	0	6	54	2	3	0	1	0	0	1	12	0	0
Avigliana	ST	0	0	7	20	3	2	0	0	0	0	0	0	0	0
Dalmazzo 1	BT/SN	564	423	0	0	71	84	0	0	0	0	0	0	0	0
	ST	1270	7547	0	0	94	115	0	0	0	0	0	0	0	0
Dalmazzo 2	BT/SN	2	1	0	0	0	0	0	0	0	0	0	0	0	0
	ST	288	200	0	0	11	7	0	0	0	0	0	0	0	0

**Table 3:** ‘*Candidatus* Liberibacter solanacearum’ infection level in psyllids collected on wild plants in 2022.

Psyllid Species	Locality	Host Plant	CLso Positive/Tested
*Heterotrioza chenopodii*	Benna (BI)	*Chenopodium album*	0/70
		*Urtica dioica*	0/2
	Borgone Susa (TO)	*Chenopodium album*	0/3
	Lagnasco (CN)	*Chenopodium album*	0/14
	S. Ambrogio (TO)	*Chenopodium album*	0/18
*Homotoma ficus*	Benna (BI)	*Ficus carica*	0/3
	Grugliasco (TO)	*Ficus carica*	0/5
*Trioza alacris*	Rivalta di Torino (TO)	*Laurus nobilis*	0/4
*Trioza urticae*	Benna (BI)	*Urtica dioica*	1/1
	S. Ambrogio (TO)	*Parietaria officinalis*	1/4
		*Urtica dioica*	0/11

**Table 4:** ‘*Candidatus* Liberibacter solanacearum’ infection level in psyllids collected from potato and carrot fields in 2023.

Host Plant	Location	Psyllid	CLso Positive/Tested			% CLso Positives
			F	M	Total	
*Solanum tuberosum*	Avigliana	*Bactericera nigricornis*	0/7	0/14	0/21	0
		*Heterotrioza chenopodii*	0/2	0/2	0/4	0
	Magnano 1	*Bactericera nigricornis*	0/5	0/8	0/13	0
		*Heterotrioza chenopodii*	0/0	0/2	0/2	0
	Magnano 2	*Bactericera nigricornis*	0/5	1/31	1/36	2.8
		*Heterotrioza chenopodii*	0/2	0/3	0/5	0
		*Aphalara freji*	0/1	0/7	0/8	0
*Daucus carota*	Dalmazzo 1	*Bactericera trigonica*	6/156	3/124	9/280	3.2
		*Heterotrioza chenopodii*	0/34	0/28	0/62	0
	Dalmazzo 2	*Bactericera trigonica*	0/78	2/139	2/217	0.9
		*Heterotrioza chenopodii*	2/11	1/6	3/17	17.6
*Chenopodium album*	Dalmazzo 1	*Heterotrioza chenopodii*	0/9	0/6	0/15	0

**Table 5:** Plant samples collected from potato and carrot fields in 2023 and tested for CLso (L = leaves, LS = leaves and stems, Ro = root, V = vein).

Locality	Plant	L	LS	Ro	V	Total	% CLso Positives
Avigliana	*Solanum tuberosum*	0/1	0/1	-	-	0/2	0
	*A. retroflexus*	0/2	-	-	-	0/2	0
	*C. arvensis*	0/1	-	-	-	0/1	0
	*G. parviflora*	0/1	-	-	-	0/1	0
Pontecurone	*Solanum tuberosum*	-	0/14	-	-	0/14	0
	*S. physalifolium*	-	0/3	-	-	0/3	0
	*C. arvensis*	-	0/1	-	-	0/1	0
	*D. carota* (wild)	-	0/1	-	-	0/1	0
Dalmazzo 1	*D. carota*	-	0/23	0/24	0/36	0/83	0
	*C. album*	-	-	-	0/9	0/9	0
Dalmazzo 2	*D. carota*	-	-	-	3/39	3/39	3.39
	*S. nigrum*	-	-	-	0/15	0/15	0
	*S. squalidus*	-	-	-	0/5	0/5	0
	*A. blitoides*	-	-	-	0/4	0/4	0
	*A. hybridus*	-	-	-	0/2	0/2	0
	*G. parviflora*	-	-	-	0/2	0/2	0

**Table 6:** Number of tested samples from asymptomatic and symptomatic plants from each of the potato and carrot fields.

Location	Plant	Asymptomatic	Symptomatic
Avigliana	*Solanum tuberosum*	(0/2)	-
Pontecurone	*Solanum tuberosum*	(0/9)	(0/5)
Dalmazzo 1	*Daucus carota*	(0/17)	(0/66)
Dalmazzo 2	*Daucus carota*	(1/25)	(2/14)

## Data Availability

Publicly available datasets were analyzed in this study (https://www.ncbi.nlm.nih.gov/), accessed on 5 May 2025. Accession numbers are reported in Figure 3. Further inquiries can be directed to the corresponding authors.

## Supplementary Materials

The following supporting information can be downloaded at: https://www.mdpi.com/article/10.3390/insects16050499/s1, Table S1: Number of psyllids collected per trap at each time point in Magnano potato fields in 2023; Table S2: Number of psyllids collected per trap at each time point in Avigliana potato field in 2023; Table S3: Number of psyllids collected per trap at each time point in Dalmazzo carrot fields in 2023.
